# Supplementary material for: Comparative efficacy and safety of caffeine citrate and aminophylline in treating apnea of prematurity: A systematic review and meta-analysis
Source: PLoS One. 2022 Sep 19;17(9):e0274882. doi: 10.1371/journal.pone.0274882 (PMC9484669; doi:10.1371/journal.pone.0274882)
Supplement: S2 Table — (DOCX) [file pone.0274882.s002.docx]

***S Table2. Search strategy.***

1. **Search criteria for PubMed:**

**#1** "Caffeine Citrate"[MeSH Terms] OR "Caffeine"[All Fields] OR "Citrates"[All Fields]

**#2** "Theophylline"[MeSH Terms] OR "Aminophylline"[All Fields] OR "Diaphylline"[All Fields] OR "Aminophyllinum"[All Fields]

**#3** "apnea"[MeSH Terms] OR "apnoea"[MeSH Terms]

**#4** "preterm infants"[MeSH Terms] OR "Infant, Premature"[All Fields] OR "Premature Infant"[All Fields] OR "Infants, Preterm"[All Fields] OR "Neonatal Prematurity"[All Fields]

**#5** #2 OR #3 OR #4

**#6** #1 AND #5  **103**

**B) Search criteria for EMBASE:**

**#1** "Caffeine Citrate"/exp OR "Caffeine"free text OR "Citrates"free text

**#2** "Theophylline"/exp OR "Aminophylline"free text OR "Diaphylline"free text OR "Aminophyllinum"free text

**#3** "apnea"/exp OR "apnoea"/exp

**#4** "preterm infants"/exp OR "Infant, Premature"free text OR "Premature Infant"free text OR "Infants, Preterm"free text OR "Neonatal Prematurity"free text

**#5** #2 OR #3 OR #4

**#6** #1 AND #5  **90**

**C) Search criteria for Web of Science:**

**#1** TS= (Caffeine Citrate OR Caffeine OR Citrates)

**#2** TS=(Theophylline OR Aminophylline OR Diaphylline OR Aminophyllinum)

**#3** TS=(apnea OR apnoea)

**#4** TS=(preterm infants ORInfant, Premature OR Premature Infant OR Infants, Preterm OR Neonatal Prematurity)

**#5** #2 OR #3 OR #4

**#6** #1 AND #2 AND #3 **37**

**D) Search criteria for the Cochrane Library:**

**#1** (Caffeine Citrate):ti,ab,kw OR (Caffeine):ti,ab,kw OR (Citrates):ti,ab,kw

**#2** (Theophylline):ti,ab,kw OR (Aminophylline):ti,ab,kw OR (Diaphylline):ti,ab,kw OR (Aminophyllinum):ti,ab,kw

**#3** (apnea):ti,ab,kw OR (apnoea):ti,ab,kw

**#4** (preterm infants):ti,ab,kw OR (Infant, Premature):ti,ab,kw OR (Premature Infant):ti,ab,kw OR (Infants, Preterm):ti,ab,kw OR (Neonatal Prematurity):ti,ab,kw

**#5** #2 OR #3 OR #4

**#6** #1 AND #5  **32**

**E) Search criteria for EBSCO:**

**#1** TX=(Caffeine Citrate OR Caffeine OR Citrates)

**#2** TX=(Theophylline OR Aminophylline OR Diaphylline OR Aminophyllinum)

**#3** TX=(apnea OR apnoea)

**#4** TX=(preterm infants ORInfant, Premature OR Premature Infant OR Infants, Preterm OR Neonatal Prematurity)

**#5** #2 OR #3 OR #4

**#6** #1 AND #5  **67**

**F) Search criteria for Scopus:**

**#1** **#1** "Caffeine Citrate" OR "Caffeine" OR "Citrates"

**#2** "Theophylline" OR "Aminophylline" OR "Diaphylline" OR "Aminophyllinum"

**#3** "apnea" OR "apnoea"

**#4** "preterm infants" OR "Infant, Premature" OR "Premature Infant" OR "Infants, Preterm" OR "Neonatal Prematurity"

**#5** #2 OR #3 OR #4

**#6** #1 AND #5  **79**
