# Supplementary material for: Comparative efficacy and safety of caffeine citrate and aminophylline in treating apnea of prematurity: A systematic review and meta-analysis
Source: PLoS One. 2022 Sep 19;17(9):e0274882. doi: 10.1371/journal.pone.0274882 (PMC9484669; doi:10.1371/journal.pone.0274882)
Supplement: S3 Table — (DOCX) [file pone.0274882.s003.docx]

***S Table3.*** ***Assessment of methodological quality by NOS.***

| Study | Selection | | | | Comparability  Comparability  of cohorts | Outcome | | | Total |
| --- | --- | --- | --- | --- | --- | --- | --- | --- | --- |
|  | Exposed cohort representativeness | Non exposed cohort selection | Ascertainment of exposure | Outcome not present at start of study |  | Assessment of outcome | Follow-up long enough | Adequacy of follow up |  |
| Lin2022 | ▲ | ▲ | ▲ |  | ▲▲ |  | ▲ | ▲ | 7 |
| Zhang2020 | ▲ | ▲ | ▲ |  | ▲▲ |  | ▲ | ▲ | 7 |
| Nagasato2018 | ▲ | ▲ | ▲ | ▲ | ▲ | ▲ | ▲ |  | 7 |
| Shivakumar2017 | ▲ | ▲ | ▲ | ▲ | ▲▲ | ▲ | ▲ |  | 8 |
| Xu2014 | ▲ | ▲ | ▲ |  | ▲ | ▲ | ▲ | ▲ | 7 |
| Skouroliakou2009 | ▲ | ▲ | ▲ | ▲ | ▲▲ | ▲ | ▲ | ▲ | 9 |
| Larsen1995 | ▲ | ▲ | ▲ | ▲ | ▲▲ | ▲ | ▲ | ▲ | 9 |
| Scanlon1992 |  |  | ▲ | ▲ | ▲▲ | ▲ | ▲ | ▲ | 7 |
